# How to improve psychiatric nosography in the XXI century: a phenomenologist”s viewpoint

**DOI:** 10.1192/j.eurpsy.2025.11

**Published:** 2025-01-27

**Authors:** Giovanni Stanghellini

**Affiliations:** 1 Department of Health Sciences, University of Florence, Florence, Italy; 2 Centro de Estudios de Fenomenologia y Psiquiatria, Universidad ‘Diego Portales’, Santiago, Chile

**Keywords:** critique of psychiatric nosography, existential knots, fragment-oriented listening, humanistic approach, structural psychopathology

## Abstract

Classifications of mental disorders reflect much more the minds of psychiatrists than the patients’ minds since these classifications are more focused on the interests of stakeholders (including governmental agencies, advocacy groups, medical insurance, and pharmaceutical companies) than on the experiences of patients. We live in times of rapid socio-cultural changes, and respective changes in the forms of mental suffering are increasingly characterized by fragmentariness and episodicity. These new forms of suffering may escape nosographic framing based on the identification of symptoms and syndromes. A paradigm shift in the psychiatric nosography is necessary. The way forward could be to enhance the ability of clinicians to grasp the “fragments” provided by patients rather than aggregations of symptoms. “Existential knots” can manifest themselves in these fragments to be used as “floating buoys” for clinical navigation, in the absence of exhaustive and detailed “maps” of the symptoms and syndromes that afflict patients. A tentative collection of these existential knots is provided, building on and extending the legacy of existential philosophy and phenomenological psychopathology.

## The social embeddedness of psychiatric nosography

In his book DSM: A History of Psychiatry”s Bible [1], sociologist and historian of psychiatry Allan V. Horowitz shows that the various editions of the Diagnostic and Statistical Manual of Mental Disorders (DSM) [2] reflect the cultural, social, and political reality of their time. The “social embeddedness” – that is, its being constrained by the environment, including institutional, political, economic, or cultural factors – of the DSM is not a negative thing in itself. On the contrary, it can be an advantage if it is understood as the attention paid by the authors of the classification of mental disorders to the social and cultural transformations of their time. It is a serious limitation when, instead of reflecting the way in which socio-cultural changes are mirrored in changes in psychopathological forms, the DSM classification of mental disorders is a “social creation” [1] that instead reflects the demands of professional or political lobbies [3]. Although in the introduction of the DSM, it is stated that the classification is not “objective,” the limitation is even more serious if the product of such intra-professional or extra-professional forces results in a widely circulated manual, advertised as the faithful mirror of mental disorders.

In short, if it is true that the various editions of the DSM reflect more the negotiations within the professional group of psychiatrists than they do the reality of mental pathology, then it is legitimate to suspect that they are very objective, and therefore scientifically relevant, document of the changes that have taken place over the last 70 years or so – not so much in the minds of patients as in the minds of psychiatrists at the top of the so-called “scientific community”. The history of the DSM indicates the manual’s deep entrenchment in the intra-professional and general sociocultural forces that impact the psychiatric profession” [1, p. 145).

## Antidotes to psychiatric nosographism: a plea for the “clinical factor” and the structural paradigm

If each era, therefore, has the DSM it deserves, why not try to image a further edition that reflects the recognition needs legitimately claimed by patients and the therapeutic and professional needs of psychiatrists working in the field? I emphasize the needs of those “working in the field,” that is, the concrete needs of those who work on the front line with “real” patients, and not in laboratories, or in those research contexts sometimes divorced from the reality of the clinic, frequented by insiders more interested in the impact factor than the clinical factor [4]. Does all entitle us to dream of a better classification of mental conditions? But let us base our dreams on some considerations dictated by a philosophical, that is, critical, reflection on the clinic.

First point: DSM diagnoses are only partially effective for “real-world” psychiatric clinics. It has long been claimed that patients often do not match diagnostic criteria and that therapeutic treatments, including biological ones, do not match nosographic criteria [5]. The correspondence between abstract criteria and actual symptoms and syndromes, and thus the clinical utility of the diagnosis, is even more limited if the assessment is aimed at other therapeutic strategies such as psychotherapeutic or rehabilitative ones. The gap widens even more if diagnostic categories are used in the relationship and dialogue with patients, for example, to inform patients of their condition, generating more misunderstandings than clarification between the two parties.

Second point: the antidote against the ineffectiveness of DSM-based nosographic diagnosis in the context of the clinic basically consists of replacing the diagnostic procedure based on ticking boxes – that is, on detecting individual symptoms with a view to a diagnosis obtained by summation – with another type of thinking and procedure that is called “structural” [6]. Structural thinking considers abnormal mental conditions not as mere aggregates of symptoms. Symptoms are a special kind of phenomena through which the hidden, yet operative (perplexing and disturbing) dimension of existence is made manifest. They are not accidental to that patient, but rather the manifestation of some implicit quintessential “core” dimension of her or his subjectivity. The overall change in the core structure of subjectivity transpires through the individual symptoms, but the specificity of the core is only graspable at a more comprehensive structural level.

This holistic approach bears little resemblance to the current atomistic operational definitions for several reasons. It goes beyond the description of isolated symptoms and the use of some of those symptoms to establish a diagnosis and aims to understand the meaning of a given set of symptoms grasping the underlying characteristic modification that keeps these symptoms meaningfully interconnected. It is capable of revealing, beyond the combinations between symptoms understood as juxtaposed elements *partes extra partes*, the reciprocal relationships, and links of meaning between the symptoms themselves. Psychopathological syndromes, in this view, are not a cacophony without order, but a melody, however strange, peculiar, and idiosyncratic. There is a method – as Shakespeare would say – in madness.

## The limitations of structural thinking in the light of fragmentation in contemporary clinic

As much as we may emphasize the importance of the “structural turn” in psychiatry, however, if we look at the presentations of psychopathological phenomena in the contemporary clinic we note a significant increase in conditions characterized by fragmentariness and the crisis of the narrative function. There is a general agreement about the hypothesis of temporal fragmentation of the self in individuals with borderline personality disorder and the close connection between their typical difficulties – for example, in interpersonal relationships – and their personal narratives highlighting their discontinuity and lack of coherence, and the association between disturbed identity and poor narrative coherence [7].

In today’s world, we see other examples of “episodic” forms of existence, marked by a diminished capacity to organize experiences into a coherent narrative, leading to existential fragmentation. Key findings emphasize the significance of anomalies in narrative identity for personality development during adolescence, especially in adolescents of the digital age [8]. We are undergoing a global shift where digital screens have evolved from mere entertainment devices to integral parts of a hybrid reality. To grasp the impact of this new hybrid reality, we must consider the activities of young people within the context of their primary developmental goal: identity formation. Constructing a personal identity is the main task of adolescence, and the ability to create a coherent life story is crucial for this process. However, identity development influenced by media experiences in the current digital ecosystem does not encourage the integration of fragmented, chronologically dispersed selves into a cohesive narrative. This contributes to an episodic and fragmented lifestyle, particularly among the post-COVID generation [8], who often have disjointed accounts of their experiences and inconsistent recollections of past events, affecting their ability to plan for the future. Clinicians are well aware of how fragmented their discourse can be.

We must prepare ourselves to witness a metamorphosis of psychopathological suffering, characterized by an increase of conditions whose brand is a profound alteration in temporality and narrative capacity, the outcome of which could be precisely fragmentariness and episodicity. The problem for the clinician may arise either from a scarcity of material due to the patients’ laconic speech or his lack of linguistic and narrative competence, or from a “superabundance” that makes it “patently impossible to establish a synthesis by assembling all the particulars” [9, p. 257]. The difficulty lies in the lack of structure of the material. From this perspective, even the most minute fragment can be an *Ansatzpunkt* (“starting point”) for exploring the patient’s subjectivity which can spread a “radiating power” – the “power to shed light in a radiating fashion” [9, p. 263]. We will have to confront these new clinical forms and prepare for a clinic that is capable of grasping the meaning of a psychopathological existence not through the reconstruction of a “totality,” but through fragments that are detached from a global structure – but not for this reason devoid of meaning. Fragments in which an attentive listener can trace in hyper-condensed form the “existential knots” with which the unfortunate existences of our patients are confronted. As explained more in detail in the next paragraph, existential knots are the diverse backdrops to a common human fate.

We must educate young clinicians on the unpredictability of the fragment. In these fragments, the existential knots are not formulated clearly, but so to speak “shine” in them. The “knot” appears and quickly disappears from the sight: it flashes and shimmers so that it hardly lets itself be glimpsed by the eye of an expert. The patient is only partially aware of the fact that that is the “node” in which her existence was trapped. Moreover, the node remains precisely at the stage of a fragment and does not unfold or configure itself in a structure, in a form of existence or a “lifeworld” that develops by articulating itself around it. Those who listen must be ready to grasp it and recognize its importance.

And if this were to be the future of our profession, then let us imagine a “nosography” constructed not as a collection of categories that assemble psychopathological symptoms (delusions, hallucinations, mood abnormalities, etc.) – but as a sylloge of limited situations, embodied by our patients, on which the becoming of their existence stops. In other words, we should try to overturn the perspective from which we usually look at mental pathology: not mental illnesses as deviations and distortions of the fundamental “normal” structures of human existence, but mental illnesses as conditions in which the existential knots or “basic concerns” that characterize the human existence – the humanity we all share – are revealed.

## A sketch of “existential knots” for clinical navigation

It is not easy to provide clinicians with a comprehensive collection of these existential “knots”. In the past, some syntheses have been attempted. Building on and extending these contributions, the list may start from Karl Jaspers’ “limit situations”, denoting the limits that are common for all persons, against which the wholeness and unity of existence may crash [10]. To these belong especially having to die, to suffer, to fight, being at the mercy of chance, and facing the inevitability of guilt. This non-systematic list of existential knots includes the fear of emptiness and meaninglessness, and the conflict between Individualization and participation, the anxiety related to isolation (the person lives in a vacuum as if there were a glass between her and the surroundings), chaos (the person lives in an unfamiliar and depersonalized world), facticity (the incapacity to shape one”s matter-of-factness) and absurdity of human existence [11]. It also includes the vertigo of freedom and the intertwining between desire and prohibition, and, finally, the quest for spontaneity and authenticity or for egocentric individual affirmation and the dialectics between existing as sentient flesh or as a visible body.

There is no unambiguous correspondence between each of these knots and the diagnoses in which traditional nosography is articulated. However, similarities can be recognized, for example between the node of guilt and the category “depression” or the node of chaos and the category “schizophrenia”. But the point is obviously not to replace one classification with another, but to identify “floating buoys” (i.e., landmarks, cardinal points) for clinical navigation in the absence of exhaustive and detailed “nautical maps”.

## Conclusions

Psychiatric classifications serve various social functions and are influenced by powerful interests. Key stakeholders include governmental agencies, medical insurance companies, advocacy groups, and pharmaceutical companies. In the 1980s, clinicians adopted the DSM to align with insurance systems, while research institutes, advocacy groups, and pharmaceutical companies used it to emphasize the need for more research, support, and treatments. However, its categories are not up to date in an era of rapid cultural change and tumultuous psychopathological metamorphosis (e.g., “episodic” psychopathology), and may not provide sufficiently valid indications for either translational research or treatment prescription – let alone for understanding patients” own ways of being in the world. The time is perhaps ripe for a change of paradigm: from the formulation of diagnostic criteria that attempt to draw a clear line between normality and pathology, to the identification of “existential knots”, proper to the *condicio humana* and not only to its psychopathological forms. Humans are inherently vulnerable and become ill when they respond inappropriately when faced with these existential knots. Can we aspire to a classification of mental conditions that truly seeks to understand the human aspects of mental suffering, rather than catering to politically motivated stakeholders who mask their interests with claims of scientific objectivity?
